# Comparative metabolomics analysis of pericarp from four varieties of *Zanthoxylum bungeanum* Maxim

**DOI:** 10.1080/21655979.2022.2108632

**Published:** 2022-10-23

**Authors:** Yonghong Cao, Miao Ren, Jianlei Yang, Lixin Guo, Yun Lin, Heng Wu, Bo Wang, Ruie Lv, Chunhui Zhang, Xutong Gong, Han Wang

**Affiliations:** a Zanthoxylum Bungeanum Research Institute, Longnan Economic Forest Research Institute, Longnan, China; b Key Laboratory of Chemistry of Northwestern Plant Resources and Key Laboratory for Natural Medicine of Gansu Province, Lanzhou Institute of Chemical Physics, Chinese Academy of Sciences, Lanzhou, China

**Keywords:** Metabolomics, different metabolites, *Zanthoxylum bungeanum* Maxim, rootstock variety

## Abstract

A qualitative and quantitative analysis of metabolites was performed by metabolomics comparation on the pericarps of four varieties of *Zanthoxylum bungeanum* Maxim. The *Zanthoxylum bunganum* as scion combined with three rootstock varieties of *Zanthoxylum piasezkii* Maxim (YJ), July *Zanthoxylum bunganum* Maxim (QJ), and August *Zanthoxylum bunganum* Maxim (BJ), at the same time *Zanthoxylum bungeanum* seedlings breeding were compared as control (MJ). A total of 1429 metabolites were identified in *Zanthoxylum bungeanum* Maxim pericarps based on chromatography and mass spectrometry dual detection platform. While the metabolites between four varieties of *Z. bungeanum* varied, there was identified 31, 15, 7, 79, 42, 19 down-regulated and 55, 50, 13, 75, 43, 27 up-regulated differential metabolites between MJ and BJ, MJ and QJ, MJ and YJ, QJ and BJ, YJ and BJ, YJ and QJ. Meanwhile, the differential metabolites composition was distinct among various varieties of *Z. bungeanum* and dominant by phenolic compounds flavonoid and phenolic acids, especially highest in varieties July *Zanthoxylum bunganum* Maxim. Highlight A comparative metabolomics analyzed in four varieties of *Zanthoxylum bungeanum* pericarp.Total 1429 metabolites were identified and mainly in flavonoid and phenolic acid.July and August *Zanthoxylum bunganum* Maxim has highest antioxidant capacity.The rootstock July *Zanthoxylum bunganum* Maxim was recommended in Loess Plateau.

## Graphical abstract

**Figure:**
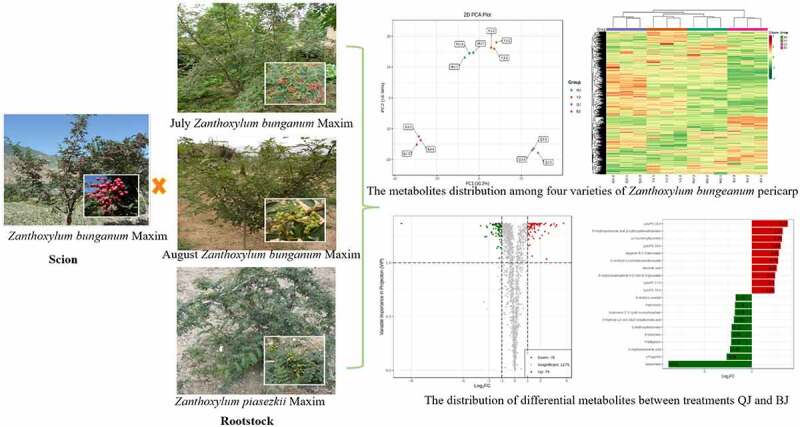

## 1. Introduction

The Rutaceae genus *Zanthoxylum* includes 250 species worldwide, among which 53 species in China and aromatic plant *Zanthoxylum bungeanum* Maxim as traditional economic crop widely distributed in southwestern China [1]. In 2018, the total production of Chinese *Zanthoxylum bungeanum* Maxim exceeded 450 thousand tons with US$18 billion market value [2]. The *Z. bungeanum* pericarp has a pungent taste and multiple active components (unsaturated fatty acids, flavonoids, alkaloids and phenols) that have attracted great attention in the area of culinary spice and medicine [3]. According to the Chinese Pharmacopoeia, *Z. bungeanum* pericarps is an ingredient in more than 30 types of prescriptions to treat pain (toothache, diarrhea and trauma) and anti-inflammatory, anti-microbial and anti-oxidative aspects [4].

For the sustainable development of *Zanthoxylum bungeanum* with broad market prospects, series of challenges such as expensive picking costs, short lifespan and weak resistance need to be overcome. In the practical operation of achieving high quality and high yield, grafting is a promising and well-developed effective approach. The selection of suitable grafting combinations (scions combined with rootstocks) is essential for plant vitality, nutrient absorption, stress resistance, yield and fruit quality [5]. The variety *Zanthoxylum bunganum* has strong capacity of tree vigor, high peel drying rate, and quality. The rootstock July *Zanthoxylum bunganum* has high and stable yield as well as strong stress resistance. The rootstock August *Zanthoxylum bunganum* has poor fruit quality and difficult to harvest while stronger in stress resistance, which is opposite to *Zanthoxylum piasezkii* Maxim. Therefore, select developed root and strong resistance variety of *Zanthoxylum bunganum* as rootstock and grafting varieties scion with excellent economic characteristics could improve plant yield and fruit quality, reduce disease occurrence and extend life duration.

Metabolites are the basis of organism phenotype, whole organism low-molecular-weight metabolites could be obtained by qualitative and quantitative metabolomics analysis, further contribute to clarify the metabolic pathway and biological processes [6]. With the technology developed, gas and liquid chromatography mass spectrometry (LC-MS and GC-MS) has developed into a powerful approach, with advantages in wide coverage, high throughput and sensitivity, and has been widely adopted in various plant physiological metabolic identification [7]. Several studies reported the differential expression of metabolites about active substance anthocyanin accumulation [6], pericarps color control and key regulatory genes [8]. Metabolites of aroma and tannin directly affect fruit quality for commodity traits in the *Zanthoxylum bungeanum* pericarps [6]. Yang et al. [9] evaluated the *Zanthoxylum bunganum* bitter compounds based on untargeted metabolomics ultra-high-performance liquid chromatography (UPLC) combined with tandem mass spectrometry. Xu et al. [10] pointed out that distinct scion and rootstock combination might be influence *Fusarium oxysporum* susceptibility by regulate stress response, carbohydrate and protein metabolism. Tietel et al. [11] reported the influence of scion/rootstock on *Citrus reticulata* metabolomics. However, limited information is available in comparative metabolomics analysis of *Zanthoxylum bungeanum* with different rootstock varieties.

In this study, a qualitative and quantitative analysis of metabolites was carried out by LC-MS and GC-MS metabolomics comparation on the pericarps of four varieties of *Zanthoxylum bungeanum*. Aimed to identify and compare the difference metabolites to determine the functional components related to nutritional health and resistance, select the optimum rootstock varieties and contributed to variety selection and genetic improvement of *Zanthoxylum bungeanum*.

## 2. Materials and methods

### 2.1. Experimental design and sample collection

The *Zanthoxylum bunganum* Maxim was cultivated in Economic Forest Research Institute, Longnan, Gansu, China. The *Zanthoxylum bunganum* as scion combined with three rootstock varieties of *Zanthoxylum piasezkii* Maxim (YJ), July *Zanthoxylum bunganum* Maxim (QJ), and August *Zanthoxylum bunganum* Maxim (BJ), at the same time *Zanthoxylum bungeanum* seedlings breeding were compared as control (MJ), three replicates for each treatment. Total sixty *Zanthoxylum bunganum* fruit randomly collected in each plant and then immediately frozen in ice box and transferred to laboratory, thereafter carefully remove *Zanthoxylum bunganum* seeds by use of scalpel and obtain pericarp stored in −80°C refrigerator.

### 2.2. Pericarp metabolites qualitative and quantitative

The pericarp samples were evenly divided into two parts and then carried out the extraction process in different platforms. For LC-MS platform, pericarp samples were first placed in lyophilizer (Scientz-100 F) for vacuum freeze-drying, and then grinded (30 Hz, 1.5 min) to powder by using grinder (MM 400, Retsch). Weigh 100 mg of the powder and dissolved in 1.2 mL of 70% methanol extract, vortex 6 times for every 30 minutes and 30 seconds once, place the sample in a 4°C refrigerator overnight, then centrifuge (12,000 rpm, 10 min) and isolated the supernatant then filtered with microporous filter (0.22 μm pore size), finally kept in sample vial for UPLC-MS/MS analysis. For GC-MS platform, pericarp samples grinded by liquid nitrogen, then vortex to mix evenly, and weigh 1 g of each sample into a headspace vial, then add saturated sodium chloride solution and 10 μL (50 μg/mL) internal standard solution. Finally, sample extracted by fully automated headspace solid-phase microextraction (HS-SPME) for GC-MS analysis.

Then perform the chromatographic mass spectrometry acquisition by ultra-performance liquid chromatography (SHIMADZU Nexera X2) and tandem mass spectrometry (Applied Biosystems 4500 QTRAP). Followed by metabolite qualitative and quantitative on LC-MS platform based on the metware database (MWDB) and the multiple reaction monitoring mode of triple quadrupole mass spectrometry. Simultaneously, qualitative and quantitative analysis of metabolite profiles on GC-MS platform based on MWGC database.

### 2.3. Data processing

The mass spectrometry metabolites qualitative analysis was carried out by Analyst 1.6.3 (AB Sciex, Framingham, MA, USA) and quantitative analysis based on the local metabolic database in LC-MS platform. The GC-MS platform qualitative analysis by using the software Qualitative Analysis Workflows B.08.00, and quantitative by MassHunter. After sample quality control analysis, principal component analysis (PCA) was performed to initially identify the overall metabolic differences between samples in each treatment and the variability within treatments. After normalized (Unit Variance Scaling) metabolite data, drawn heat map and hierarchical cluster by R software. Repetitive correlations were assessed by Pearson’s correlation coefficient using R software. The partial least squares discriminant analysis (PLS-DA) was performed by SIMCA v14.1 to transform the original data and mean centering, then use the MetaboAnalyst R package OPLSR. Anal function for analysis, maximizing the distinction between treatments and contributed to differential metabolites observation [12].

Differential metabolite screening was based on the OPLS-DA results, and from the obtained multivariate analysis of the Variable Importance in Projection (VIP≥ 1) combined with the difference fold value (Fold Change ≥ 2 and Fold Change ≤ 0.5) of the univariate analysis. Based on the differential fold changes in the quantitative information of metabolites in each treatment, draw differential metabolite bar graphs and volcano plots by using R to identified the content difference metabolites and the statistical significance. Then, identified the original contents of differential metabolites and normalized, then clustering heatmap by the R software Complex Heatmap package. Finally, based on the differential metabolite results, performed kyoto encyclopedia of genes and genomes (KEGG) pathway enrichment analysis [13].

## 3. Results

### 3.1. Overall metabolites and principal component analysis

A total of 1429 metabolites were identified in *Zanthoxylum bungeanum* Maxim pericarps based on chromatography and mass spectrometry dual detection platform. In detail, comprised of 288 flavonoids, 202 phenolic acids, 138 lipids, 121 alkaloids, 102 amino acids and derivatives, 94 terpenoids, 82 organic acids, 76 lignans and coumarins, 62 nucleotides and derivatives, 49 ester, 17 ketone, 16 heterocyclic compound, 14 hydrocarbons, 12 alcohol, 12 tannins, 11 aldehyde, 7 aromatics, 5 halogenated hydrocarbons, 2 amine, 2 acid, 1 phenol, 1 sulfur compounds, 1 quinones and 114 others. The significantly different metabolites between the varieties of *Z. bungeanum* were listed in Table 1. The identified metabolites contributed to the isolation and identification of *Z. bungeanum* pericarp functional compounds for valuable utilization.

**Table 1.** The significantly differential metabolite between four varieties of *Zanthoxylum bungeanum* pericarp.

Treatments	All significantly differential metabolites	Down regulated	Up regulated
MJ vs BJ	86	31	55
MJ vs QJ	65	15	50
MJ vs YJ	20	7	13
QJ vs BJ	154	79	75
YJ vs BJ	85	42	43
YJ vs QJ	46	19	27

MJ: *Zanthoxylum bungeanum* seedlings breeding, YJ: *Zanthoxylum bunganum* as the scion variety and rootstock with *Zanthoxylum piasezkii* Maxim, QJ: *Zanthoxylum bunganum* as the scion variety and rootstock with July *Zanthoxylum bunganum* Maxim, and BJ: *Zanthoxylum bunganum* as the scion variety and rootstock with August *Zanthoxylum bunganum* Maxim.

**Figure 1.**
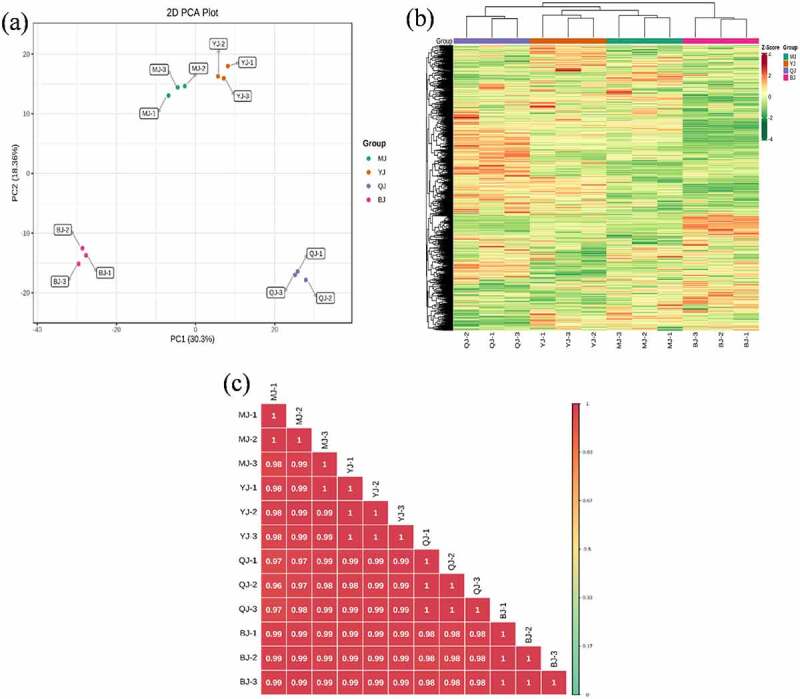
The principal component analysis (PCA) (Figure 1a), heat map (Figure 1b) and correlation (Figure 1c) among four varieties of *Zanthoxylum bungeanum* pericarp.

**Figure 2.**
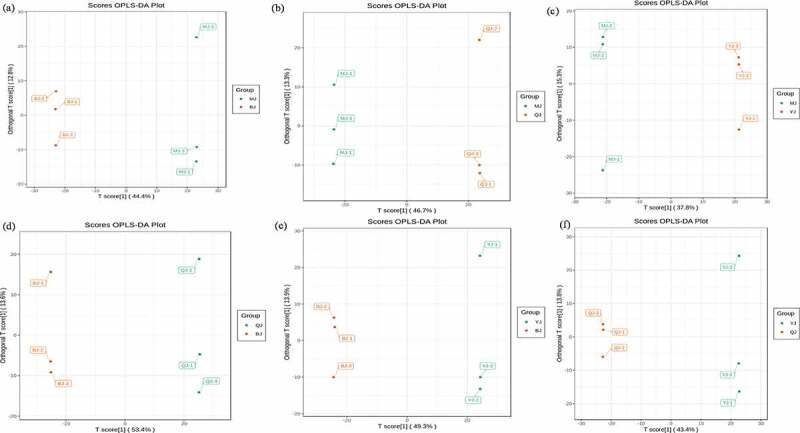
The orthogonal partial least square discriminant analysis (OPLS-DA) score between treatment MJ and BJ (Figure 2a), MJ and QJ (Figure 2b), MJ and YJ (Figure 2c), QJ and BJ (Figure 2d), YJ and BJ (Figure 2e), YJ and QJ (Figure 2f).

**Figure 3.**
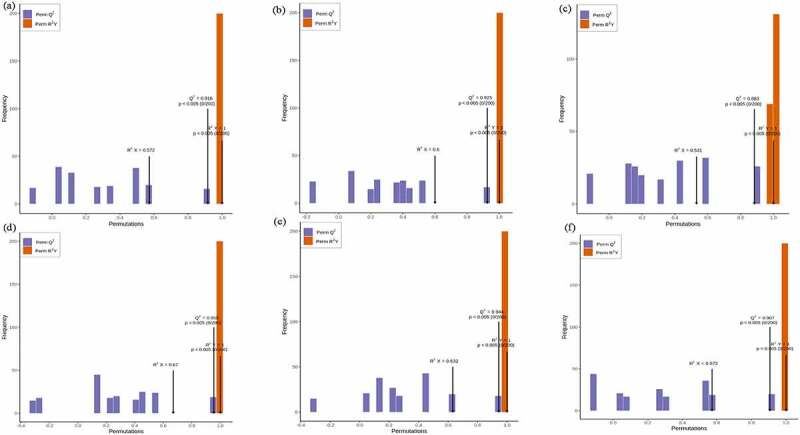
The orthogonal partial least square discriminant analysis model validation diagram between treatment MJ and BJ (Figure 3a), MJ and QJ (Figure 3b), MJ and YJ (Figure 3c), QJ and BJ (Figure 3d), YJ and BJ (Figure 3e), YJ and QJ (Figure 3f).

The overall treatment PCA scores of the mass spectrometry data for each treatment quality control samples showed that two principal components (PC1 and PC2) accounted for 30.3% and 18.36%. Four varieties of *Z. bungeanum* were obviously separated while three replicates were closely clustered in each treatment (Figure 1a) and the heat map also obviously gathered as four categories (Figure 1b). The correlation between MJ and YJ, MJ and QJ were 0.98–0.99, 0.96–0.97, while both was 0.99 between MJ and BJ, YJ and BJ, YJ and QJ (Figure 1c). At the same time, the OPLS-DA plot showed that the T1 score was 44.4, 46.7, 37.8, 53.4, 49.3, and 43.4% with orthogonal T score 12.8, 13.3, 15.3, 13.6, 13.9, and 13.8% between MJ and BJ, MJ and QJ, MJ and YJ, QJ and BJ, YJ and BJ, YJ and QJ (Figure 2). The OPLS-DA model demonstrated R^2^X = 0.6, 0.6, 0.5, 0. 7, 0.6, 0.6, R^2^Y = 1 and Q^2^ > 0.90 (Figure 3), which indicated the repeatability and reliability of the experiment and the clear variation of metabolites in the four varieties of *Z. bungeanum*. Accordingly, rootstock selection strongly effects the metabolite profile of *Z. bungeanum*.

### 3.2. Differential metabolite screening

**Figure 4.**
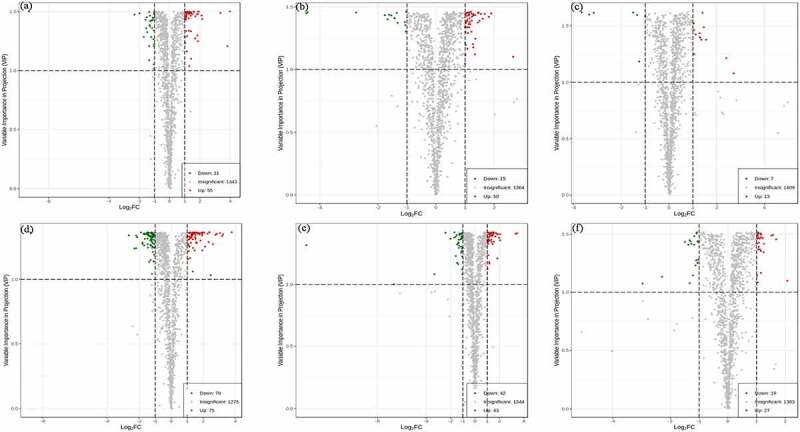
The volcano plots based differential metabolite contents between treatment MJ and BJ (Figure 4a), MJ and QJ (Figure 4b), MJ and YJ (Figure 4c), QJ and BJ (Figure 4d), YJ and BJ (Figure 4e), YJ and QJ (Figure 4f).

**Figure 5.**
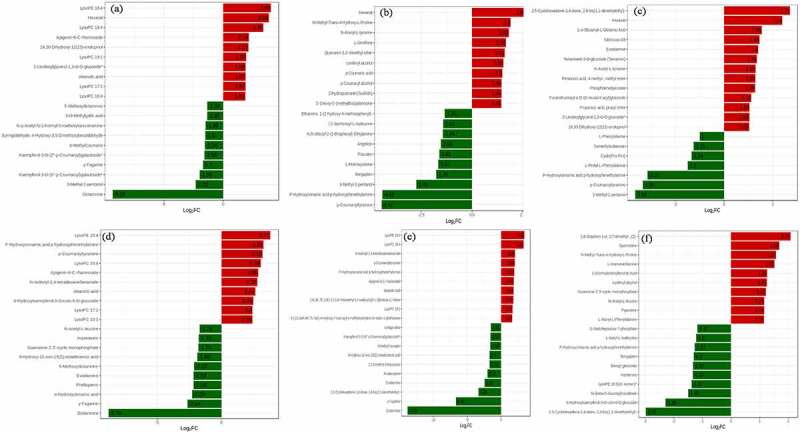
The difference multiples histogram based top ten differential metabolite contents in treatment MJ and BJ (Figure 5a), MJ and QJ (Figure 5b), MJ and YJ (Figure 5c), QJ and BJ (Figure 5d), YJ and BJ (Figure 5e), YJ and QJ (Figure 5f).

The differential metabolites between *Zanthoxylum bungeanum* pericarp of different rootstock-scion combinations demonstrated in volcano plots. There were identified 31, 15, 7, 79, 42, 19 down-regulated and 55, 50, 13, 75, 43, 27 up-regulated differential metabolites between MJ and BJ, MJ and QJ, MJ and YJ, QJ and BJ, YJ and BJ, YJ and QJ (Figure 4). Meanwhile, the difference of metabolites in MJ and BJ, QJ and BJ, YJ and BJ greater than MJ and QJ, MJ and YJ, YJ and QJ. In detail, for treatments MJ and BJ, the top ten up-regulated differential metabolites were lysope 18:4, hexanal, lysopc 18:4, apigenin-6-c-rhamnoside, 24,30-dihydroxy-12(13)-enolupinol, lysopc 19:1, 2-linoleoylglycerol-1,3-di-o-glucoside, oleanolic acid, lysopc 17:1, and lysopc 16:4 (log_2_FC = 3.99, 3.83, 3.36, 2.16, 2.12, 1.92, 1.88, 1.87, 1.87, 1.85). The top ten down-regulated differential metabolites included dictamnine, 3-methyl-1-pentanol, kaempferol-3-o-(6”-p-coumaroyl) galactoside, γ-fagarine, kaempferol-3-o-(2”-p-coumaroyl) galactoside, 6-methylcoumarin, 4-hydroxy-3,5-dimethoxybenzaldehyde, n-γ-acetyl-n-2-formyl-5-methoxykynurenamine, 3-o-methylgallic acid, and 5-methoxydictamnine (log_2_FC = −9.28, −2.32, −1.98, −1.7, −1.58, −1.54, −1.5, −1.48, −1.35, −1.34) (Figure 5a).

For treatments MJ and QJ, top ten up-regulated differential metabolites included hexanal, n-methyl-trans-4-hydroxy-l-proline, n-acetyl-l-tyrosine, l-ornithine, quercetin-3,3’-dimethyl ether, coniferyl alcohol, p-coumaric acid, p-coumaryl alcohol, dihydroquercetin (taxifolin), and 5’-deoxy-5’-(methylthio) adenosine (log_2_FC = 2.66, 1.9, 1.82, 1.68, 1.61, 1.55, 1.5, 1.46, 1.46, 1.45), top ten down-regulated differential metabolites included p-coumaroyltyramine, p-hydroxycinnamic acid p-hydroxyphenethylamine, 3-methyl-1-pentanol, bergapten, l-homocysteine, psoralen, angelicin, n-dibutyl-2-(2-thiophenyl)-ethylamine, jasmonoyl-l-isoleucine, and ethanone (log_2_FC = −4.47, −4.42, −2.75, −1.76, −1.67, −1.62, −1.54, −1.44, −1.43, −1.35) (Figure 5b). For treatments MJ and YJ, top ten up-regulated differential metabolites included 2,5-cyclohexadiene-1,4-dione, hexanal, l-α-glutamyl-l-glutamic acid, sibiricose a5, evodiamine, tamarixetin-3-o-glucoside (tamarixin), n-acetyl-l-tyrosine, pentanoic acid, phosphoenolpyruvate, furanofructosyl-α-d-(6-mustard acyl) glucoside, propanoic acid, 2-linoleoylglycerol-1-3-di-o-glucoside, and 24,30-dihydroxy-12(13)-enolupinol (log_2_FC = 2.71, 2.4, 1.55, 1.46, 1.4, 1.36, 1.29, 1.29, 1.28, 1.12, 1.05, 1.05, 1.03). The top seven down-regulated differential metabolites included 3-methyl-1-pentanol, p-coumaroyltyramine, p-hydroxycinnamic acid p-hydroxyphenethylamine, l-prolyl-l-phenylalanine, cyclo (pro-pro), demethylsuberosin, and l-phenylalanine (log_2_FC = −3.66, −3.35, −3.16, −1.5, −1.34, −1.25, −1) (Figure 5c).

For treatments QJ and BJ, top ten up-regulated differential metabolites included lysope 18:4, p-hydroxycinnamic acid p-hydroxyphenethylamine, p-coumaroyltyramine, lysopc 18:4, apigenin-6-c-rhamnoside, n-isobutyl-2,4-tetrade canedienamide, oleanolic acid, 6-hydroxykaempferol-3-o-rutin-6-o-glucoside, lysopc 17:1, and lysopc 19:1 (log_2_FC = 3.77, 3.24, 3.18, 3.04, 2.85, 2.76, 2.61, 2.46, 2.4, 2.38). The top ten down-regulated differential metabolites included dictamnine, γ-fagarine, α-hydroxycinnamic acid, phellopterin, evodiamine, 5-methoxydictamnine, 9-hydroxy-12-oxo-15(z)-octadecenoic acid, guanosine 3’,5’-cyclic monophosphate, imperatorin, and n-acetyl-l-leucine (log_2_FC = −8.76, −2.64, −2.29, −2.18, −2.18, −2.12, −1.89, −1.79, −1.78, −1.72) (Figure 5d). For treatments YJ and BJ, top ten up-regulated differential metabolites included lysope 18:4, lysopc 18:4, n-isobutyl-2,4-tetradecanedienamide, p-coumaroyltyramine, p-hydroxycinnamic acid p-hydroxyphenethylamine, apigenin-6-c-rhamnoside, oleanolic acid, (1r,3e,7e,11 r)-1,5,5,8-tetramethyl-12-oxabicyclo [9.1.0] dodeca-3,7-diene, lysopc 19:1, and ethanone (log_2_FC = 3.45, 3.33, 2.06, 2.06, 1.97, 1.88, 1.84, 1.81, 1.71, 1.67). The top ten down-regulated differential metabolites included dictamnine, γ-fagarine, 2,5-cyclohexadiene-1,4-dione, 2,6-bis (1,1-dimethylethyl), evodiamine, rutaecarpine, 2,2-dimethyl-3-heptanone, 9-hydroxy-12-oxo-15(z)-octadecenoic acid, 6-methylcoumarin, kaempferol-3-o-(6”-p-coumaroyl) galactoside, and isofagaridine (log_2_FC = −13.84, −6.66, −3.34, – 2.42, −2.02, −1.71, −1.7, −1.58, −1.58, −1.51) (Figure 5e). For treatments YJ and QJ, top ten up-regulated differential metabolites included 2,6-octadien-1-ol, 3,7-dimethyl, spermidine, n-methyl-trans-4-hydroxy-l-proline, l-homomethionine, 2-(formylamino) benzoic acid, coniferyl alcohol, guanosine 3’,5’-cyclic monophosphate, n-acetyl-l-leucine, piperidine, and l-alanyl-l-phenylalanine (log_2_FC = 2.07, 1.68, 1.57, 1.52, 1.26, 1.25, 1.21, 1.16, 1.15, 1.15). The top ten down-regulated differential metabolites included 2,5-cyclohexadiene-1,4-dione, 2,6-bis (1,1-dimethylethyl), 6-hydroxykaempferol-3-o-rutin-6-o-glucoside, n-(beta-d-glucosyl) nicotinate, lysope 18:3 (2 n isomer), hordenine, benzyl glucoside, bergapten, p-hydroxycinnamic acid p-hydroxyphenethylamine, l-seryl-l-isoleucine, and d-sedoheptuiose 7-phosphate (log_2_FC = −2.97, −2.29, −1.49, −1.37, −1.33, −1.33, −1.3, −1.27, −1.21, −1.17) (Figure 5f).

**Figure 6.**
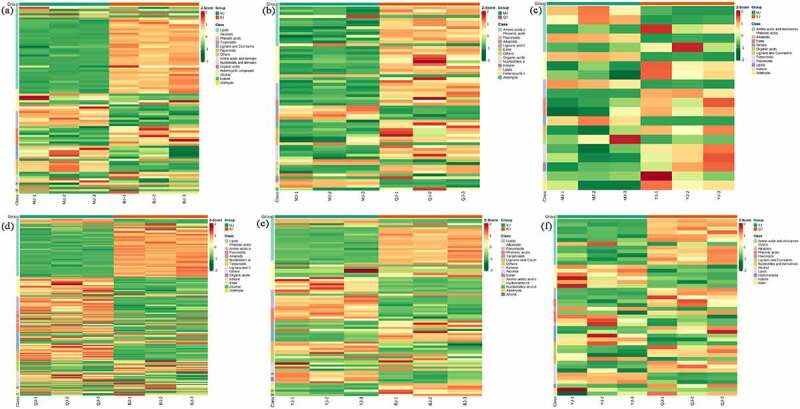
The clustering heatmap based on differential metabolite contents between treatment MJ and BJ (Figure 6a), MJ and QJ (Figure 6b), MJ and YJ (Figure 6c), QJ and BJ (Figure 6d), YJ and BJ (Figure 6e), YJ and QJ (Figure 6f).

The clustering heatmap based on relative content of differential metabolites displayed that compare MJ and BJ, BJ dominant by lipids (lysope, lysopc), terpenoids (4-hydroxybenzoyl-1-o-(6”-o-galloyl) glucoside, oleanolic acid, cis-nerolidol), while MJ dominant by alkaloids (5-methoxydictamnine, γ-fagarine), phenolic acids (3-o-methylgallic acid), flavonoids (kaempferol-3-o-(2”-p-coumaroyl) glucoside) and lignans and coumarins (esculetin, imperatorin, isoimperatorin, phellopterin) (Figure 6a). When compared to MJ and QJ, QJ mainly distributed in amino acids and derivatives (l-glutamine, l-lysine, l-homomethionine), phenolic acids (cinnamic acid, p-coumaryl alcohol, hydrocinnamic acid, p-coumaric acid, (e)-3-(3,4-dihydroxyphenyl) acrylaldehyde, α-hydroxycinnamic acid, 2-(formylamino) benzoic acid, ethylparaben, coniferaldehyde), flavonoids (dihydroquercetin (taxifolin) kumatakenin, tamarixetin, isorhamnetin, quercetin-3,3’-dimethyl ether), and ester (isobornyl formate, benzenepropanoic acid, pentanoic acid, 4-methyl-, methyl ester). While MJ dominant by amino acids and derivatives (l-homocysteine), lignans and coumarins (psoralen, angelicin) (Figure 6b). Compare MJ and YJ, YJ dominant by phenolic acids (sibiricose a5, furanofructosyl-α-d-(6-mustard acyl) glucoside), ester (pentanoic acid, propanoic acid), organic acids phosphoenolpyruvate, while MJ richer in amino acids and derivatives (l-prolyl-l-phenylalanine, p-hydroxycinnamic acid), and alkaloids (p-coumaroyltyramine) (Figure 6c).

Compare QJ and BJ, BJ dominant by lipids (lysopc, lysope), terpenoids (24,30-dihydroxy-12(13)-enolupinol,12,13-dihydroursolic acid, cadina-1(10),6,8-triene), organic acids (3-methyl-2-oxobutanoic acid, argininosuccinic acid), while QJ dominant by phenolic acids (isovanillin, caffeic acid), amino acids and derivatives (l-homocystine), flavonoids (kaempferol-3-o-glucoside (astragalin)), nucleotides and derivatives (cytidine, guanosine 5’-monophosphate) (Figure 6d). Compare YJ and BJ, YJ dominant by flavonoids (azaleatin (5-o-methylquercetin), quercetin-3,3’-dimethyl ether), ketone (sibiricose a5), ketone (2,5-cyclohexadiene-1,4-dione, 2,6-bis(1,1-dimethylethyl),2,2-dimethyl-3-heptanone). While BJ dominant in lysopc, terpenoids (oleanolic acid,24,30-dihydroxy-12(13)-enolupinol) (Figure 6e). Compare YJ and QJ, YJ dominant by maltotriose and d (+)-melezitose o-rhamnoside, while QJ mainly distributed in amino acids and derivatives (l-homomethionine, l-leucyl-l-leucine, n-acetyl), flavonoids (3-o-acetylpinobanksin), phenolic acids (p-coumaric acid, 2-(formylamino) benzoic acid) (Figure 6f). Therefore, the up-regulated and down-regulated differential metabolites as well as dominant metabolites varied in *Zanthoxylum bunganum* Maxim with distinct kind of rootstock varieties.

### 3.3. Differential metabolite kyoto encyclopedia of genes and genome enrichment

**Figure 7.**
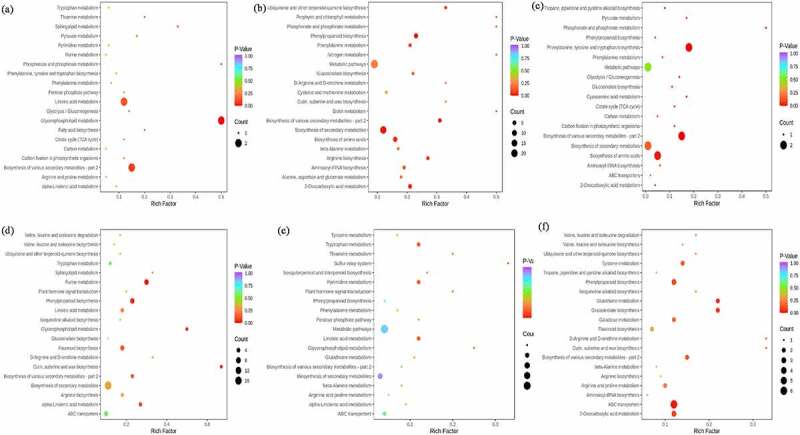
The kyoto encyclopedia of genes and genomes (KEGG) pathway enrichment between treatment MJ and BJ (Figure 7a), MJ and QJ (Figure 7b), MJ and YJ (Figure 7c), QJ and BJ (Figure 7d), YJ and BJ (Figure 7e), YJ and QJ (Figure 7f).

The KEGG database is a powerful tool for metabolic pathway and metabolic network, which contributes to the whole network from gene expression information and metabolite content [13]. In this study, differential metabolites enrichment analysis was performed between *Zanthoxylum bungeanum* pericarp of different rootstock-scion combinations. For treatment MJ and BJ, the differential metabolites total includes 86 compounds with 31 down regulation (7 phenolic acids, 6 lignans and coumarins and 6 alkaloids) and 55 up regulations (35 lipids and 8 terpenoids), involved 23 pathways and mainly distributed in metabolic pathways (71.43%), followed by biosynthesis of secondary metabolites (21.43%), glycerophospholipid metabolism (14.29%) and linoleic acid metabolism (14.29%), of which only glycerophospholipid metabolism was significantly up regulated (p-value <0.05) (Figure 7a). For treatment MJ and QJ, the differential metabolites total includes 65 compounds with 15 down regulation (3 lignans and coumarins, 2 amino acids and derivatives and 2 alkaloids) and 50 up regulations (12 amino acids and derivatives, 12 phenolic acids and 5 ester), involved 51 pathways and predominant by metabolic pathways (77.42%), followed by biosynthesis of secondary metabolites (58.06%) and biosynthesis of amino acids (22.58%), especially 2-oxocarboxylic acid metabolism, biosynthesis of amino acids and arginine biosynthesis were significantly regulated (p-value <0.05) (Figure 7b). For treatment MJ and YJ, the differential metabolites total includes 20 kinds of compounds with 7 down regulation (3 amino acids and derivatives, 1 phenolic acids and alkaloids) and 13 up regulations (2 amino acids and derivatives, 2 phenolic acids and ester), involved 19 pathways and phenylalanine, tyrosine and tryptophan biosynthesis, metabolic pathways, biosynthesis of secondary metabolites, and biosynthesis of various secondary metabolites-part 2 account for 100%, of which phenylalanine, tyrosine and tryptophan biosynthesis, biosynthesis of various secondary metabolites-part 2, phosphonate and phosphinate metabolism, biosynthesis of amino acids, cyanoamino acid metabolism, pyruvate metabolism, glycolysis/ gluconeogenesis, citrate cycle (tca cycle), carbon fixation in photosynthetic organisms, and glucosinolate biosynthesis were significantly regulated (p-value <0.05) (Figure 7c).

For treatment QJ and BJ, the differential metabolites total includes 154 compounds with 79 down regulation (13 amino acids and derivatives, 15 phenolic acids, 14 flavonoids, and 10 alkaloids) and 75 up regulations (8 terpenoids, and 45 lipids), involved 33 pathways and mainly distributed in metabolic pathways (67.5%), biosynthesis of secondary metabolites (42.5%), and purine metabolism (15%), whereas purine metabolism, cutin, suberine and wax biosynthesis, and phenylpropanoid biosynthesis shown p-value <0.05 (Figure 7d). For treatment YJ and BJ, the differential metabolites total includes 85 compounds with 42 down regulation (7 phenolic acids, 8 flavonoids, and 9 alkaloids) and 43 up regulations (8 terpenoids, and 17 lipids), involved 20 pathways and mainly in metabolic pathways (62.5%) and biosynthesis of secondary metabolites (25%) but no significant pathways (p-value >0.05) (Figure 7e). For treatment YJ and QJ, the differential metabolites total includes 46 compounds with 19 down regulation (6 others, 2 flavonoids, and 2 phenolic acids) and 27 up regulations (11 amino acids and derivatives, 3 phenolic acids, and alkaloids), involved 26 pathways includes metabolic pathways (61.1%), biosynthesis of secondary metabolites (33.3%), and abc transporters (33.3%), of which only abc transporters significantly regulated (p-value <0.05) (Figure 7f).

## 4. Discussion

The differential metabolites up-regulated or down-regulated and dominant metabolites varied in *Zanthoxylum bungeanum* pericarp with distinct varieties of rootstock-scion. Total 1429 metabolites were identified and mainly distributed in polyphenols compounds of flavonoid and phenolic acid. The polyphenols were one of the largest secondary metabolites in plants, which positively respond to abiotic stress and pathogenesis resistance and immunity enhancement [14]. Many polyphenols have been reported, such as phenolic acid, flavonoid and proanthocyanidins [6]. Primarily, flavonoid was specialized metabolites that act in widely physiological processes and responses to biological and abiotic stresses such as ultraviolet radiation and cold resistance, which are critical to response the unique natural environment of the Loess Plateau [15]. Ravishankar et al. [16] also reported that flavonoids have diverse biological and pharmacological activities which contribute to anticancer and anti-inflammatory due to strong antioxidant compounds. Additionally, flavonoids related with bitter or astringent and act as important contributors to coloring substances, especially metabolites of quercetin contributed to the green color of *Zanthoxylum bungeanum* [17]. Zheng et al. [6] found that flavonoids were the predominant metabolites to regulate redness of *Zanthoxylum bungeanum* pericarp. More than 8000 flavonoids with pharmacological properties have been identified and the high content of rutin, quercitrin and hesperidin have strong antioxidant activities that enhanced oxidative stress of plants and drought resistance [18]. The highest flavonoids activity demonstrated in treatment QJ and BJ, followed by YJ and BJ, especially beneficial substance isorhamnetin only observed in QJ, quercetin only existed in QJ and YJ, and coumarin with therapeutic potential identified in BJ and QJ. The significant distinct composition and content of flavonoids resulted in various antioxidant activities and resistance during four *Zanthoxylum bungeanum* cultivars might be attributed to differences in rootstocks.

In addition, phenolic acids usually existed with polysaccharides in plant cell walls with antioxidant effects [19], the composition and activity varied among different *Zanthoxylum bunganum* varieties and highest activity in QJ and BJ, MJ and QJ, which were related to the antioxidant and enzyme inhibitory activities [20]. Therefore, the differential metabolic varied with distinct rootstocks of *Zanthoxylum bunganum*, and the differences in the antioxidant activities are attributed to the changes of phenolic compounds, the overall antioxidant activity strongest in QJ and BJ treatments [21]. Moreover, the alkaloids were widely used for medical purposes due to bioactive components with pharmacological value (such as anti-inflammatory and analgesic) and essential in plant stress and pathogen defense, significantly upregulated in treatment QJ and BJ [22]. Organic acids were key intermediates in carbohydrate catabolism and involved in a variety of metabolic pathways, include stress defense and carbon storage as well as biosynthesis of primary/secondary metabolites, such as azelaic acid, salicylic acid and abscisic acid participated in plant defense against harmful pathogens or pests [23,24], but only identified in BJ and YJ.

The terpenoids contribute essential role between plant and insect, pathogen and plant interactions [25–27], and the increased ratio of esters include alcohols and aldehydes was attributed by the amino acids Strecker reaction and phospholipid-induced lipid degradation [28,29]. Thus, rootstock-scion combination is capable to regulate amino acid and lipid content to control the flavor characteristics of *Zanthoxylum bungeanum*. Besides, free amino acids were involved in the aroma substances formation and enhanced plant stress resistance by induce physiological mechanisms [28–30]. The sensitivity of amino acids to stress was varied in distinct varieties of *Zanthoxylum bungeanum*, especially the most sensitive metabolic was QJ and BJ. Overall, the metabolism of rootstock July *Zanthoxylum bunganum* Maxim and scion *Zanthoxylum bunganum* combination was activated in physiological, biochemical and molecular adaptations with the highest antioxidant capacity and stress resistance.

## 5. Conclusion

The metabolites component and content in *Zanthoxylum bungeanum* pericarp of different rootstock-scion combinations were varied, dominant by polyphenols compounds of flavonoids and phenolic acids and highest in treatment QJ. The stress sensitivity of the active ingredient varied in different varieties of *Zanthoxylum bungeanum*, especially most sensitive in QJ and BJ. Finally, the metabolism of rootstock July *Zanthoxylum bunganum* Maxim combined with scion *Zanthoxylum bunganum* was activated in physiological, biochemical and molecular adaptations, contributed to highest antioxidant capacity and stress resistance in response to the unique natural environment of the Loess Plateau.

## Novelty statement

This study qualitative and quantitative analysis of metabolites on the pericarps of four varieties of *Zanthoxylum bungeanum* Maxim by metabolomics comparative. Rootstock selection strongly effects the pericarp metabolite profile of *Z. bungeanum* varieties. The primarily for polyphenols included flavonoids and phenolic acids were predominant compounds. Overall, the metabolism of rootstock July *Zanthoxylum bunganum* Maxim and scion *Zanthoxylum bunganum* combination was activated in physiological, biochemical and molecular adaptations, contributed to highest antioxidant capacity and stress resistance to response the unique natural environment of the Loess Plateau.

## References

[1] Wang K, Meng X, Chai T, et al. Chemical constituents from the fruits of *Zanthoxylum bungeanum* and their chemotaxonomic significance. Biochem Syst Ecol. 2021;99:104356.

[2] Jing N, Wang M, Gao M, et al. Color sensory characteristics, nutritional components and antioxidant capacity of *Zanthoxylum bungeanum* Maxim. as affected by different drying methods. Ind Crop Prod. 2021;160:113167.

[3] Sun X, Zhang D, Zhao L, et al. Antagonistic interaction of phenols and alkaloids in Sichuan pepper (*Zanthoxylum bungeanum*) pericarp. Ind Crop Prod. 2020;152:112551.

[4] Deng S, Rong H, Tu H, et al. Molecular basis of neurophysiological and antioxidant roles of Szechuan pepper. Biomed Pharmacother. 2019;112:108696. 30818139 10.1016/j.biopha.2019.108696

[5] Sousa A, Silva E, Filho M, et al. Metabolic responses to drought stress and rehydration in leaves and roots of three Citrus scion/rootstock combinations. Sci Hortic. 2022;292:110490.

[6] Zheng T, Zhang Q, Su K, et al. Transcriptome and metabolome analyses reveal the regulation of peel coloration in green, red Chinese prickly ash (*Zanthoxylum L*.). Food Chem Mol Sci. 2020;1:100004. 10.1016/j.fochms.2020.100004 PMC8991852 35415618

[7] Li W, Wen L, Chen Z, et al. Study on metabolic variation in whole grains of four proso millet varieties reveals metabolites important for antioxidant properties and quality traits. Food Chem. 2021;357:129791. 33895687 10.1016/j.foodchem.2021.129791

[8] Chen X, Wang W, Wang C, et al. Quality evaluation and chemometric discrimination of *Zanthoxylum bungeanum* Maxim leaves based on flavonoids profiles, bioactivity and HPLC-fingerprint in a common garden experiment. Ind Crop Prod. 2019;134:225–233.

[9] Yang Q, Mei X, Wang Z, et al. Comprehensive identification of non-volatile bitter-tasting compounds in *Zanthoxylum bungeanum* Maxim. by untargeted metabolomics combined with sensory-guided fractionation technique. Food Chem. 2021;347:129085. 33493837 10.1016/j.foodchem.2021.129085

[10] Xu D, Yuan H, Tong Y, et al. Comparative proteomic analysis of the graft unions in Hickory (Carya cathayensis) provides insights into response mechanisms to grafting process. Front Plant Sci. 2017;8:676. 28496455 10.3389/fpls.2017.00676 PMC5406401

[11] Tietel Z, Srivastava S, Fait A, et al. Impact of scion/rootstock reciprocal effects on metabolomics of fruit juice and phloem sap in grafted Citrus reticulata. PLoS One. 2020;15:1–17. 10.1371/journal.pone.0227192 PMC6953815 31923191

[12] Wang F, Huang Y, Wu W, et al. Metabolomics analysis of the peels of different colored citrus fruits (*Citrus reticulata* cv. ‘Shatangju’) during the maturation period based on UHPLC-QQQ-MS. Molecules. 2020;25:396. 10.3390/molecules25020396 PMC7024170 31963595

[13] Xiao J, Gu C, He S, et al. Widely targeted metabolomics analysis reveals new biomarkers and mechanistic insights on chestnut (*Castanea mollissima Bl*.) calcification process. Food Res Int. 2021;141:110128. 33641995 10.1016/j.foodres.2021.110128

[14] Terahara N. Flavonoids in foods: a review. Nat Prod Commun. 2015;10:521–528. 25924542

[15] Zeng X, Yuan H, Dong X, et al. Genome-wide dissection of Co-selected UV-B responsive pathways in the UV-B adaptation of Qingke. Mol Plant. 2020;13:112–127. 31669581 10.1016/j.molp.2019.10.009

[16] Ravishankar D, Rajora A, Greco F, et al. Flavonoids as prospective compounds for anti-cancer therapy. Int J Biochem Cell B. 2013;45:2821–2831. 10.1016/j.biocel.2013.10.004 24128857

[17] Li P, Zhu Y, Lu M, et al. Variation patterns in the content of glycosides during green tea manufacturing by a modification-specific metabolomics approach: enzymatic reaction promoting an increase in the glycosidically bound volatiles at the pan firing stage. Food Chem. 2019;279:80–87. 30611515 10.1016/j.foodchem.2018.11.148

[18] Yang C, Yang H, Xu Q, et al. Comparative metabolomics analysis of the response to cold stress of resistant and susceptible *Tibetan hulless barley* (*Hordeum distichon*). Phytochemistry. 2020;174:112346. 32229337 10.1016/j.phytochem.2020.112346

[19] Liu J, Zhang X, Tian J, et al. Multiomics analysis reveals that peach gum colouring reflects plant defense responses against pathogenic fungi. Food Chem. 2022;383:132424. 35182869 10.1016/j.foodchem.2022.132424

[20] Shen R, Ma Y, Jiang L, et al. Chemical composition, antioxidant, and antiproliferative activities of nine Chinese proso millet varieties. Food Agr Immunol. 2018;29:625–637.

[21] Hui W, Wang J, Ma L, et al. Identification of key genes in the biosynthesis pathways related to terpenoids, alkaloids and flavonoids in fruits of *Zanthoxylum armatum*. Sci Hortic. 2021;290:110523.

[22] Ke J, Cheng J, Luo Q, et al. Identification of two bitter components in *Zanthoxylum bungeanum* Maxim. and exploration of their bitter taste mechanism through receptor hTAS2R14. Food Chem. 2020;338:127816. 32818866 10.1016/j.foodchem.2020.127816

[23] Berens M, Berry H, Mine A, et al. Evolution of hormone signaling networks in plant defense. Ann Rev Phytopathol. 2017;55:401–425. 28645231 10.1146/annurev-phyto-080516-035544

[24] Wang F, Chen L, Chen H, et al. Analysis of flavonoid metabolites in citrus peels (*Citrus reticulata* “Dahongpao”) using UPLC-ESI-MS/MS. Molecules. 2019;24:2680. 10.3390/molecules24152680 PMC6696472 31344795

[25] Stringlis I, de Jonge R, Pieterse C. The age of coumarins in plantmicrobe interactions. Plant Cell Physiol. 2019;60:1405–1419. 31076771 10.1093/pcp/pcz076 PMC6915228

[26] Wang H, Hua J, Jiang Y, et al. Influence of fixation methods on the chestnut-like aroma of green tea and dynamics of key aroma substances. Food Res Int. 2020;136:109479. 32846562 10.1016/j.foodres.2020.109479

[27] Wang H, Hua J, Yu Q, et al. Widely targeted metabolomic analysis reveals dynamic changes in non-volatile and volatile metabolites during green tea processing. Food Chem. 2021;363:130131. 34120048 10.1016/j.foodchem.2021.130131

[28] Zou S, Wu J, Shahid M, et al. Identification of key taste components in loquat using widely targeted metabolomics. Food Chem. 2020;323:126822. 32334307 10.1016/j.foodchem.2020.126822

[29] Kaigongi M, Lukhoba C. The chemosystematics of the genus *Zanthoxylum* L. (Rutaceae) in Kenya. Biochem Syst Ecol. 2021;98:104319.

[30] Tian J, Ma Y, Tian L, et al. Comparative physiology and transcriptome response patterns in cold-tolerant and cold-sensitive varieties of *Zanthoxylum bungeanum*. Maxim. Ind Crop Prod. 2021;167:113562.

